# Nurse-Initiated Improvement for Documentation of Penicillin Adverse Drug Reactions in Pediatric Urgent Care Clinics

**DOI:** 10.3390/children12081087

**Published:** 2025-08-19

**Authors:** Elizabeth Monsees, Diane Petrie, Rana E. El Feghaly, Sarah Suppes, Brian R. Lee, Megan Whitt, Amanda Nedved

**Affiliations:** 1 Patient Care Services Research, Children’s Mercy Kansas City, Kansas City, MO 64108, USA; eamonsees@cmh.edu; 2 Department of Pediatrics, University of Missouri-Kansas City, Kansas City, MO 64108, USA; relfeghaly@cmh.edu (R.E.E.F.); blee@cmh.edu (B.R.L.); 3 Department of Pediatrics, Children’s Mercy Kansas City, Kansas City, MO 64108, USA; 4 Division of Infectious Diseases, Children’s Mercy Kansas City, Kansas City, MO 64108, USA; drpetrie@cmh.edu; 5 Division of Clinical Pharmacology, Children’s Mercy Kansas City, Kansas City, MO 64108, USA; slsuppes@cmh.edu; 6 Division of Health Services and Outcomes Research, Children’s Mercy Kansas City, Kansas City, MO 64108, USA; 7 Division of Urgent Care, Children’s Mercy Kansas City, Kansas City, MO 64108, USA; mlwhitt@cmh.edu

**Keywords:** penicillin, antimicrobial stewardship, electronic health record, documentation, adverse drug reaction, health equity, quality improvement

## Abstract

**Highlights:**

**What are the main findings?**

- Nurse-initiated interventions improved penicillin allergy label documentation.
- Improved documentation of reactions did not increase triage time.

**What is the implication of the main finding?**

- Nurse engagement in initiatives can offer new perspectives and contribute to success
- Future efforts may focus on engaging families in penicillin allergy discussions.

**Abstract:**

**Background/Objective:** Penicillin allergy labels (PALs) contribute to broad-spectrum antibiotic use. Thorough documentation can help prescribers identify and remove unnecessary PALs. We aimed to improve documentation of PALs in three pediatric urgent care (PUC) clinics, using a nurse-initiated quality improvement (QI) approach. **Methods:** QI interventions included a survey to assess prescriber and nurse confidence, an online educational module, and an algorithm to aid in clarifying PALs. We measured the percentage of PALs with a clarified reaction severity as our primary outcome using annotated control charts. Descriptive and inferential statistics evaluated survey responses between nurses and prescribers. **Results:** Clarified PAL reaction severity had a sustained upward shift from 58.5% to 63.3% following implementation of our interventions. Of 129 nurses and prescribers, 87 (67.4%) respondents completed the survey. Prescribers and nurses reported feeling knowledgeable about PALs but experienced different challenges to clarifying PAL documentation. Prescribers reported time pressures as a barrier to PAL clarification more often than nurses (IQR [3, 4], *p* = 0.001). Nurses reported higher confidence in ability to document a PAL compared to prescribers (IQR [3.25, 5], *p* = 0.010). Respondents requested family education and practice guidance to aid PAL clarification. No consistent differences were noted in PAL documentation by sociodemographic characteristics. **Conclusions:** The nurse-initiated QI approach demonstrated improved PAL documentation in PUCs. Engaging nurses in antibiotic stewardship initiatives can provide new perspectives and broaden the approach to intervention design and implementation. Future efforts should focus on improving electronic health record and interprofessional workflow processes to build on these improvements.

## 1. Introduction

Penicillins (PCN), part of the beta-lactam group, are the most common antibiotics prescribed and the recommended treatment for many common infections [1,2]. Approximately 5–10% of children have a reported PCN allergy label (PAL), with 75% of children receiving this label by 3 years of age [2,3,4]. Studies report that after appropriate evaluation, approximately 95% of children with PALs do not have a clinically significant adverse drug reaction (ADR) to PCN (acute onset IgE-mediated or delayed-onset T-cell-mediated hypersensitivity) [3]. PALs increase exposure to broad-spectrum antimicrobials, which can lead to increased healthcare cost due to more expensive antibiotic use, prolonged hospital admissions, decreased therapeutic effectiveness, and increased risk of ADRs to other medications [5,6]. PALs have also been associated with increased risk of antibiotic-resistant infections [7]. Because of these undesired patient effects and associated healthcare costs, antimicrobial stewardship advocates have made identifying and delabeling erroneous PALs a priority [8].

Direct antibiotic challenge testing is an effective way to formally evaluate and remove erroneous PALs. However, these evaluations often occur in subspecialty clinics, which may not be accessible to all families. Studies in both adult and pediatric populations demonstrated that non-specialists, including nurses, pharmacists, and prescribers, can successfully delabel PALs in the inpatient setting by focusing on clarifying the reaction and risk stratification [9,10,11]. Additionally, a study of children who had their PAL removed in the primary care setting—either by chart review or by retrial of a penicillin antibiotic—tolerated PCN as often as those delabeled by an allergist. Furthermore, investigators in this study found that approximately 50% of PALs had an allergy severity of “unable to classify” documented [12]. A recent study demonstrated nurses can appropriately identify patients with low and high-risk PCN allergies highlighting the important role of nurses in improving the accuracy of PALs [13].

We found similar percentages (41.5%) of unclarified allergy severities in our pediatric urgent care (PUC) clinics. This missing and incomplete documentation of PALs likely contributed to unnecessary avoidance of PCNs. Additionally, partial documentation likely represented a broader issue that reported PALs were not being thoroughly interrogated prior to adding them to the electronic health record (EHR). Clarifying the reported PAL and documenting the details in the EHR at the time of labeling help avoid unnecessary PALs and identify opportunities to remove erroneous labels in the future. In response to this identified gap in care, we designed a quality improvement (QI) study to evaluate the impact of nurse-led interventions aimed at enhancing and clarifying reaction severity.

## 2. Methods

### 2.1. Context

We conducted a QI study in three free-standing PUCs located throughout a Midwest metropolitan area. These clinics are part of a pediatric, academic hospital system and have more than 90,000 pediatric encounters annually. The PUCs are staffed by general pediatricians, advanced practice registered nurses (APRN)s, and registered nurses.

**Figure 1:**
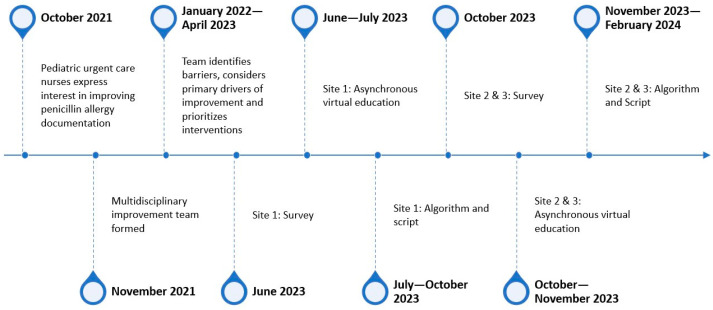
Study timeline.

After identifying PAL documentation challenges, PUC nurses championed the formation of a multidisciplinary QI team in January 2022. The team included PUC nurses and prescribers, in addition to experts in antimicrobial stewardship and QI methodology. From January 2022 through April 2023, we used QI tools to understand the problem, explore potential causes of unclear/incomplete ADR documentation, consider interventions that would improve ADR documentation, and evaluate the impact using iterative Plan-Do-Study-Act (PDSA) cycles. Barriers identified through a fishbone diagram included nurse discomfort with questioning parent or caregiver PAL report, unfamiliarity with the delabeling process or our organization’s resources for formal evaluation of PAL, time constraints, and the EHR allowing incomplete documentation of PALs (e.g., no hard stop for blank fields for severity or description). We considered primary drivers to improve documentation of PALs, which included education, prescriber and nurse engagement, and EHR support. Using change ideas identified in the driver diagram, we used a prioritization matrix to evaluate which interventions would have the greatest impact. After piloting the initial interventions at a single site and identifying opportunities for improvement through PDSA cycles, outlined below, we deployed the same format at the two other PUC locations (Figure 1).

### 2.2. Interventions

For our first PDSA cycle, we used the Research Electronic Data Capture (REDCap) application [14] to develop an 18-question, anonymous survey using a 5-point Likert scale (1 = strongly disagree, 5 = strongly agree) to evaluate nurse and prescriber confidence in assessing, documenting, and responding to PALs to inform tool development. The survey included questions on PCN and patient safety (4), PAL categories (3), PAL documentation (4), and treatment options (3)—all scored using a 5-point Likert-scale. The remaining questions included 4 respondent demographic questions and one free-text option to comment on the PUC PAL documentation process. We used unit-based listservs to distribute the survey and sent 2 invitation reminders during the 2 weeks the survey was available.

For our second PDSA cycle, we developed a voluntary, asynchronous, interactive virtual educational module targeting nurses and prescribers using the Rise (Articulate Global LLC, New York, NY, USA) application. The module reviewed several types of reactions that may receive a PAL (e.g., parental or religious preference, side effect, hypersensitivity), how to assess and document PALs in the EHR, criteria needed for a true hypersensitivity reaction, and the process of delabeling patients through referral to our subspecialty clinics. Participants could stop and return to the sessions that took approximately 30 min to complete in total. We offered continuing education credit for nurses who completed the module. The module was available to nurses and prescribers for 4 weeks during a period of low patient census.

Our third and final PDSA cycle targeted improving nurse comfort with clarifying PALs with families and relaying information to prescribers about erroneous PALs. We developed an algorithm to assist nurses in stratifying patient reactions by type and severity, then recommended management based on assessed risk. The algorithm also provided a standardized script nurses could use when communicating with patients and families or prescribers (Supplemental Material). A member from the hospital system’s family advisory council reviewed the language for clarity and content. Following feedback and guidance from nurses and prescribers, we posted the algorithm in various locations through the PUCs for 3 months following deployment of the interactive educational module.

### 2.3. Study of the Intervention

We created a monthly EHR report of PUC encounters with an active PAL at the time of presentation. We defined a PAL as those with a reported ADR to ampicillin, penicillin, Amoxil^®^ (amoxicillin), Augmentin^®^ (amoxicillin-clavulanate), oxacillin, nafcillin, and Unasyn^®^ (ampicillin-sulbactam). We retrospectively obtained 11 months of baseline data (July 2022–May 2023) and then monthly data from June 2023 to June 2024. We considered June 2023 to February 2024 as the intervention period when we actively implemented PDSA cycles. We considered March 2024 to June 2024 as the post-intervention period, as no new interventions occurred during this time. We collected PAL characteristics of severity as a binary “Known” (e.g., mild, moderate, severe) or “Unknown” at the time of documentation. We also collected duration of nurse triage. Additionally, we collected patient demographics, including race, preferred language, and insurance type, to determine whether documentation varied by these characteristics. We created a virtual dashboard using Power BI (Microsoft Corporation; Redmond, WA, USA) for data visualization and trended data by month and shared data with PUC staff during monthly team meetings.

### 2.4. Measures

We defined our primary outcome as the proportion of PUC encounters with PAL that had a “Known” severity. For our process measure, we evaluated survey responses by nurses and prescribers and the frequency of completed educational modules by discipline, including the percentage of completion by practice location. Our balancing measure evaluated the median duration of time to complete the triage note as a proxy for PAL reconciliation time. We also evaluated if there were differences in the percentage of clarified reaction severity for PALs in PUC encounters among sociodemographic variables. Due to the limitations of its paper format, we could not capture algorithm usage.

### 2.5. Statistical Analysis

We created a proportions (p) control chart for our outcome measure using QI Macros 2023 software (KnowWare International, Inc., Denver, CO, USA, version 2023.07.1). We used standard Shewhart rules to identify special cause variation to shift the mean and control limits [15]. We compared proportions of “Known” PAL severity among different sociodemographic groups, stratified by the 3 study time points (i.e., baseline, intervention, post-intervention) using Pearson’s chi-square test. All comparisons by demographics were completed using Python statistical software (version 3.9.2).

We used descriptive statistics to evaluate survey responses between nurses and prescribers, basic demographic information (e.g., educational level), and by PUC location. We performed a concept analysis to code open-ended survey responses and grouped codes into themes using Microsoft Excel (Microsoft 365 subscription, version 16.014326.21008).

## 3. Results

### 3.1. Patient Demographics

During the study period, we included 14,084 PUC encounters with a documented PAL. We evaluated 6760 encounters during the baseline period (July 2022–May 2023), 5090 encounters during the intervention period (June 2023–February 2024), and 2234 encounters during the post-intervention period (March 2024–June 2024). The patient population across the 3 intervention periods was similar with the most common demographics reported as White (69%), commercially insured (54.5%), and English-speaking (97.1%) (Table 1).

During the baseline period, we identified differences in the proportion of PALs with a “Known” severity for race/ethnicity and insurance (Table 2).

The difference in proportion for race/ethnicity persisted during the intervention period but diminished in the post-intervention period. We identified differences in the proportion of PALs with a “Known” severity for language in the post-intervention period that had not been present during the other time periods. However, this is likely related to the small number of encounters that reported Spanish or Other language during this time.

### 3.2. Primary Outcome and Balancing Measure

**Figure 2:**
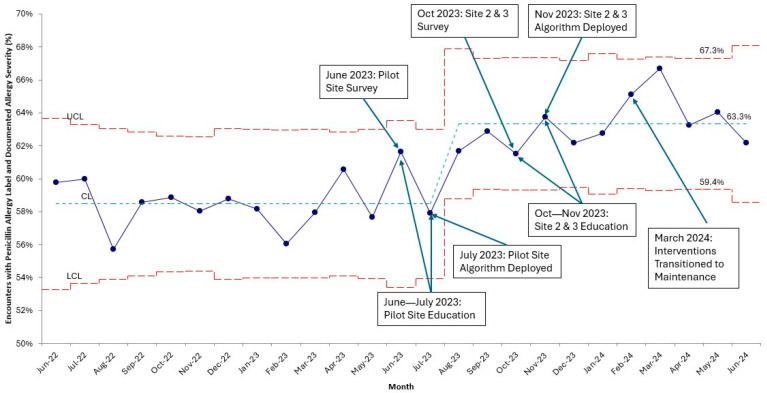
Annotated proportion (P) control chart of the percentage of encounters with documented PAL severity. Shewhart Control Chart Rules identified special cause variation in August 2023 with 8 consecutive points above the center line.

At baseline, 58.5% of PUC encounters with PAL had a “Known” reaction severity. We saw an upward shift of the center line in August 2023 to 63.3% after deployment of the education module at the pilot PUC. This change was sustained throughout the deployment of interventions to the other 2 PUCs (October 2023) and remained stable as the interventions transitioned to a maintenance post-intervention period (March 2024–June 2024) (Figure 2).

We did not see a change in the median triage documentation time for nursing, which remained stable at 3 min during the baseline, intervention, and post-intervention periods.

### 3.3. Process Measure

In total, 129 PUC nurses and prescribers (97 nurses, 9 APRNs, and 23 physicians) completed the education module. Completion rate across the 3 PUCs was similar. More than half of respondents (n = 80, 62%) reported 11 or more years of experience. Twenty-four (19%) self-identified as having a leadership role (e.g., director, assistant manager, core charge nurse).

### 3.4. Survey

Our survey had a 35% response rate with 87 nurses and prescribers indicating they worked primarily in the PUC either as full time or float/temporary team members. Many respondents (40%, n = 35) were highly experienced and had been in practice at our institution for >15 years (Table 3).

Knowledge of PALs and safety was favorable across disciplines. Although both disciplines responded confidently that patients with PAL can safely take PCN, prescribers responded more confidently than nurses (prescribers’ median = 5 [Interquartile Range (IQR):4,4] vs. nurses’ median = 4 [4, 4], *p* = 0.003). Nurses and prescribers agreed that PALs change over time (prescribers’ median = 4 [3.75, 4] vs. nurses’ median = 4 [4, 5]), *p* = 0.16. Nurses responded more confidently in their ability to appropriately document a PAL in the EHR (prescribers’ median = 4 [3, 4] vs. nurses’ median = 4 [3.25, 5] *p* = 0.010), particularly when a parent described the presentation of a side effect. Prescribers reported that the pace of the PUC influenced their ability to reconcile allergies and side effects more than nurses (prescribers’ median = 4 [4, 5] vs. nurses’ median = 3 [2, 4], *p* = 0.001). Both prescribers and nurses responded less confidently regarding awareness of available resources to evaluate PALs at our institution (prescribers’ median = 3 [2, 4], nurses’ median = 3 [2, 4]; *p*= 0.987) and comfort with prescribing for an antibiotic with an ADR listed in the EHR (prescribers’ median = 3 [2, 4], nurses’ median = 3 [2, 4]; *p*= 0.405) (Table 4).

Thirteen respondents (15%) commented on their experiences with the PAL documentation process. Common themes included a desire for more information on the referral process for formal evaluation of PALs (n = 5) and the creation of family education to augment discussions occurring during clinical care (n = 4), particularly when “it’s ok to still give [PCN] despite a reported ‘allergy’” or about “the hereditary risk (or lack thereof) of antibiotic allergies” as future interventions. Both prescribers and nurses expressed continued challenges with role clarity. A physician with more than 15 years of experience shared, “One would think a primary care physician would be better equipped to discuss this, tease out all the details of the reaction, and refer a patient for penicillin allergy testing if deemed necessary rather than doing this through an urgent care”. A nurse with more than 15 years of experience shared, “I never know if it is in the scope of the bedside nurse to discuss with the family adverse drug reaction vs. side effect, and do not feel comfortable changing what is reported”.

## 4. Discussion

This multisite nurse-led QI study partnered with prescribers and antibiotic stewardship experts to design and implement several interventions to improve PAL reconciliation and documentation in PUCs. We identified four key findings to advance interprofessional stewardship efforts. First, we saw an increase in the percentage of PAL encounters with a “Known” reaction severity following our interventions without increasing triage documentation time. Second, nurses and prescribers expressed a mutual desire for additional information on PALs and delabeling processes, including Supplementary Materials to improve interactions with families during the clinical visit. Third, we did not detect a difference in documentation by sociodemographic characteristics as our population was largely homogenous. However, it is important to consider health equity in designing stewardship interventions. Finally, we add to the evidence that nurses are not only a valuable interdisciplinary partner, but they can also be successful leaders of antibiotic stewardship and QI endeavors.

In our study, PUC nurses and prescribers acknowledged the importance of PAL documentation in their survey responses. Clarifying the reported PAL when families initially report an ADR may avoid unnecessary PALs from occurring. Documenting the details of the reaction helps identify opportunities to remove erroneous labels in the future. Yet, in practice, over 50% of our PUC encounters with a PAL did not have their reaction severity documented at baseline. Our approach to this gap in care shared similarities with Hampton et al.’s QI initiative to improve PAL documentation among hospitalized children. In their study, the completeness of PALs among children admitted to the hospital medicine service improved from 20% to 64% following education, implementation of a clinical pathway that included risk stratification, and EHR changes [11].

Nurses initiated this study because they wanted to address documentation challenges when families report side effects or preference as a reason to avoid PCN exposure. In our previous work, nurses expressed discomfort with clarifying the PAL with family or relaying concerns to prescribers, despite clear understanding of side effects vs. true allergies [16]. These perceptions are consistent with perceptions reported in other studies that nurses prefer tools to facilitate discussions or to use as a practice reference [17,18]. Despite our survey results indicating that most nurses and prescribers were highly experienced, both groups requested additional education on their respective roles for documenting and risk-stratifying PALs. Specifically, they wanted more information on referring patients with PALs to subspecialty clinics for formal evaluation. This aligns with previous reports that families and clinicians may be reluctant to rely solely on historical report to remove PALs [19,20]. The educational module, algorithm, and script attempted to address these reported barriers. However, we acknowledge that additional opportunities exist to broaden nurse and prescriber awareness of best practices for PAL documentation and delabeling practices including guidance on communicative and behavioral approaches when side effects are identified. Additionally discipline-specific EHR interventions that consider the unique role of each discipline in the documentation and review of PALs may be helpful.

Previous studies reported differences in rates of PALs among children of differing socioeconomic backgrounds, with Black patients having lower rates than White patients [3,21,22]. Patients with government and self-pay insurance also have lower rates of reported PALs than those with commercial insurance [21,22]. We stratified our data by sociodemographic variables to evaluate if differences in PAL documentation contributed to the pattern of inequitable PAL percentages previously reported. Our results identified differences in the documentation of reaction severity by race/ethnicity, insurance, and language during different periods of the study. However, the differences were not consistent and the absolute differences in percentage between the different groups were considered small. While our study did not identify a consistent pattern of inequity, ensuring that PAL documentation does not differ across family populations is an important educational consideration in promoting equitable antibiotic stewardship [23].

Engaging nurses in antibiotic stewardship collaborations has grown in recent years with many studies demonstrating nursing contributions as integral to the success of initiatives [18,24,25,26,27,28]. We considered the successful leadership of our nurses to engineer this interdisciplinary collaboration as an example for other antibiotic stewardship endeavors to be a strength of this study. In our study, nurses developed the survey, partnered with content experts in infectious diseases providing input on the relevant content for the education module and algorithm, promoted the educational modules to their colleagues, posted the algorithm in high-usage areas, shared data and feedback with participants, and disseminated findings at the hospital’s Patient Care Services Research Grand Rounds. By sharing their insights and expertise on ADR documentation, the front-line nurses provided a unique approach to antibiotic stewardship. We encourage continued collaboration of all team members for the development of innovative stewardship initiatives.

### Limitations

Due to the inherent limitations of a retrospective chart review of the EHR, we could not determine the accuracy of documentation for PALs including reaction severity. Additionally, any clinical employee within our system could modify the ADR documentation. Our data represents PUCs within a single organization which limits the generalizability of our results. However, our results are consistent with other studies that demonstrate challenges in clarifying PAL documentation and inaccuracies in PAL classification even when clarified [12,29,30]. This pervasive challenge reported across multiple healthcare organizations underscores the importance for systematic improvements to accurately document PALs in the EHR.

## 5. Conclusions

Using interventions initiated by nurses and informed by front-line interdisciplinary collaboration, we increased the documentation of PAL severity in PUC encounters without a negative impact on patient workflow. Ensuring accurate documentation is an important next step in the evaluation and management of patients with PALs. Future work will focus on continuing our partnership between nurses and prescribers to develop communication strategies with families regarding PALs, improve the evaluation process for identifying patients who can safely be delabeled either via history or referral for antibiotic challenge testing, and ensure equitable opportunities for PAL evaluation.

## Figures and Tables

**Table 1:** Patient demographics.

	Overall	Baseline Period	Intervention Period	Post-Intervention Period
**Total Encounters**	14,084	6760	5090	2234
***Race/Ethnicity, n (%)***				
Asian	257 (1.8)	111 (1.6)	98 (1.9)	48 (2.1)
Black	1096 (7.8)	516 (7.6)	399 (7.8)	181 (8.1)
Hispanic	2003 (14.2)	970 (14.3)	718 (14.1)	315 (14.1)
Multiracial	651 (4.6)	329 (4.9)	239 (4.7)	83 (3.7)
White	9724 (69.0)	4652 (68.8)	3517 (69.1)	1555 (69.6)
Other	243 (1.7)	118 (1.7)	88 (1.7)	37 (1.7)
Unknown/Declined	110 (0.8)	64 (0.9)	31 (0.6)	15 (0.7)
***Insurance, n (%)***				
Commercial	7674 (54.5)	3622 (53.6)	2837 (55.7)	1215 (54.4)
Medicaid	5527 (39.2)	2776 (41.1)	1920 (37.7)	831 (37.2)
Self-pay	438 (3.1)	150 (2.2)	177 (3.5)	111 (5.0)
Other/Unknown	445 (3.2)	212 (3.1)	156 (3.1)	77 (3.4)
***Language, n (%)***				
English	13,678 (97.1)	6560 (97.0)	4953 (97.3)	2165 (96.9)
Spanish	295 (2.1)	139 (2.1)	106 (2.1)	50 (2.2)
Other	111 (0.8)	61 (0.9)	31 (0.6)	19 (0.9)

**Table 2:** Proportion of documented versus unknown reaction severity for penicillin allergy labels by race/ethnicity, insurance, and language.

	Baseline Period N = 6760			Intervention Period N = 5090			Post-Intervention Period N = 2234		
	Documented	Unknown	*p* Value	Documented	Unknown	*p* Value	Documented	Unknown	*p* Value
***Race/Ethnicity, n (%)***			**0.005**			**<0.001**			**0.349**
Asian	57 (1.4)	54 (2.0)		47 (1.4)	51 (2.8)		28 (1.9)	20 (2.7)	
Black	301 (7.3)	215 (8.1)		246 (7.5)	153 (8.4)		110 (7.4)	71 (9.4)	
Hispanic	594 (14.5)	376 (14.2)		468 (14.3)	250 (13.7)		216 (14.6)	99 (13.2)	
Multiracial	179 (4.4)	150 (5.6)		178 (5.4)	61 (3.4)		59 (4.0)	24 (3.2)	
White	2845 (69.3)	1807 (68.0)		2252 (68.9)	1265 (69.5)		1032 (69.6)	523 (69.5)	
Other	83 (2.0)	35 (1.3)		61 (1.9)	27 (1.5)		25 (1.7)	12 (1.6)	
Unknown/Declined	45 (1.1)	19 (0.7)		18 (0.6)	13 (0.7)		12 (0.8)	3 (0.4)	
**Insurance, n (%)**			**0.037**			**0.599**			**0.830**
Commercial	2207 (53.8)	1415 (53.3)		1812 (55.4)	1025 (56.3)		811 (54.7)	404 (53.7)	
Medicaid	1692 (41.2)	1084 (40.8)		1250 (38.2)	670 (36.8)		552 (37.2)	279 (37.1)	
Self-pay	96 (2.3)	54 (2.0)		114 (3.5)	63 (3.5)		70 (4.7)	41 (5.5)	
Other/Unknown	109 (2.7)	103 (3.9)		94 (2.9)	62 (3.4)		49 (3.3)	28 (3.7)	
**Language, n (%)**			**0.616**			**0.769**			**0.033**
English	3977 (96.9)	2583 (97.3)		3178 (97.2)	1775 (97.5)		1427 (96.3)	738 (98.1)	
Spanish	90 (2.2)	49 (1.8)		71 (2.2)	35 (1.9)		38 (2.6)	12 (1.6)	
Other	37 (0.9)	24 (0.9)		21 (0.6)	10 (0.5)		17 (1.1)	2 (0.3)	

**Table 3:** Characteristics of survey respondents.

	Prescribers (n = 40)	Nurses (n = 47)
***Primary Urgent Care Clinic, n (%)***		
A	13 (32.5)	14 (29.8)
B	11 (27.5)	11 (23.4)
C	14 (35.0)	22 (46.8)
Missing	2 (5.0)	0 (0.0)
***Time since last clinical degree, n (%)***		
<1 year	0 (0.0)	1 (2.2)
1–5 years	5 (12.5)	8 (17.4)
6–10 years	10 (25.0)	10 (21.7)
11–15 years	9 (22.5)	7 (15.2)
>15 years	16 (40.0)	20 (43.5)
***Worked at institution, n (%)***		
<5 years	9 (22.5)	10 (21.7)
5–10 years	10 (25.0)	12 (26.1)
11–15	7 (17.5)	3 (6.5)
>15 years	14 (35.0)	21 (45.6)

**Table 4:** Number of Responses and Level of Agreement on a 5-point Likert Scale (1 = Strongly Disagree, 5 = Strongly Agree) to Statements Related to Penicillin Allergy and Safety.

	Prescribers (n = 40)		Nurses (n = 47)		
	Respondents	Median Level of Agreement [IQR]	Respondents	Median Level of Agreement [IQR]	*p* Value
I am confident in my ability to identify delayed reactions to antibiotics based on timing of symptoms after ingestion of the antibiotic.	40	4 [3, 4]	46	3 [3, 4]	0.416
Many patients who think they are allergic to penicillin can safely take penicillin.	39	5 [4, 5]	46	4 [4, 4]	0.003
I am knowledgeable about the risks of avoiding penicillin in patients that have a documented penicillin allergy.	39	4 [4, 5]	45	4 [3, 4]	0.056
I can distinguish between common pediatric conditions that are often misinterpreted as a penicillin allergy (i.e., viral rash, vomiting/diarrhea).	40	4 [3, 4]	47	4 [3, 4]	0.880
I am aware that penicillin allergy sensitivities can change over time.	40	4 [3.75, 4]	47	4 [4, 4.5]	0.160
I am able to identify factors associated with true allergic reactions.	40	4 [4, 4]	45	4 [4, 4]	0.980
I am aware of the types of penicillin allergy challenges that [our institution] offers.	40	3 [2, 4]	45	3 [2, 4]	0.987
I feel confident in my ability to appropriately document an adverse drug reaction in the electronic health record, even when a family describes side effects.	40	4 [3, 4]	46	4 [3.25, 5]	0.010
My documentation of adverse drug reactions influences future antibiotic prescribing.	40	4 [4, 5]	44	4 [4, 5]	0.356
Time pressures (e.g., patient flow) influence my ability to reconcile between allergy and side effect.	39	4 [3, 4]	45	3 [2, 4]	0.001
Perceived parent expectations influence my ability to reconcile between allergy and side effects.	40	4 [3.75, 4]	44	4 [3, 4]	0.529
I feel confident continuing to administer or prescribe an antibiotic in the setting of a reported adverse drug reaction.	40	3 [2, 4]	44	3 [2, 4]	0.409
I feel confident in my ability to talk with families about antibiotic side effects and reactions.	40	4 [3, 4]	46	4 [3, 4]	0.033

## Data Availability

The dataset will not be available.

## Supplementary Materials

The following supporting information can be downloaded at: https://www.mdpi.com/article/10.3390/children12081087/s1, Algorithm and scripting developed to aid nurses in risk stratification and discussion with families and prescribers regarding penicillin allergy evaluation.
