# Supplementary figures and images for: Nurse-Initiated Improvement for Documentation of Penicillin Adverse Drug Reactions in Pediatric Urgent Care Clinics
Source: Children (Basel). 2025 Aug 19;12(8):1087. doi: 10.3390/children12081087 (PMC12384498; doi:10.3390/children12081087)

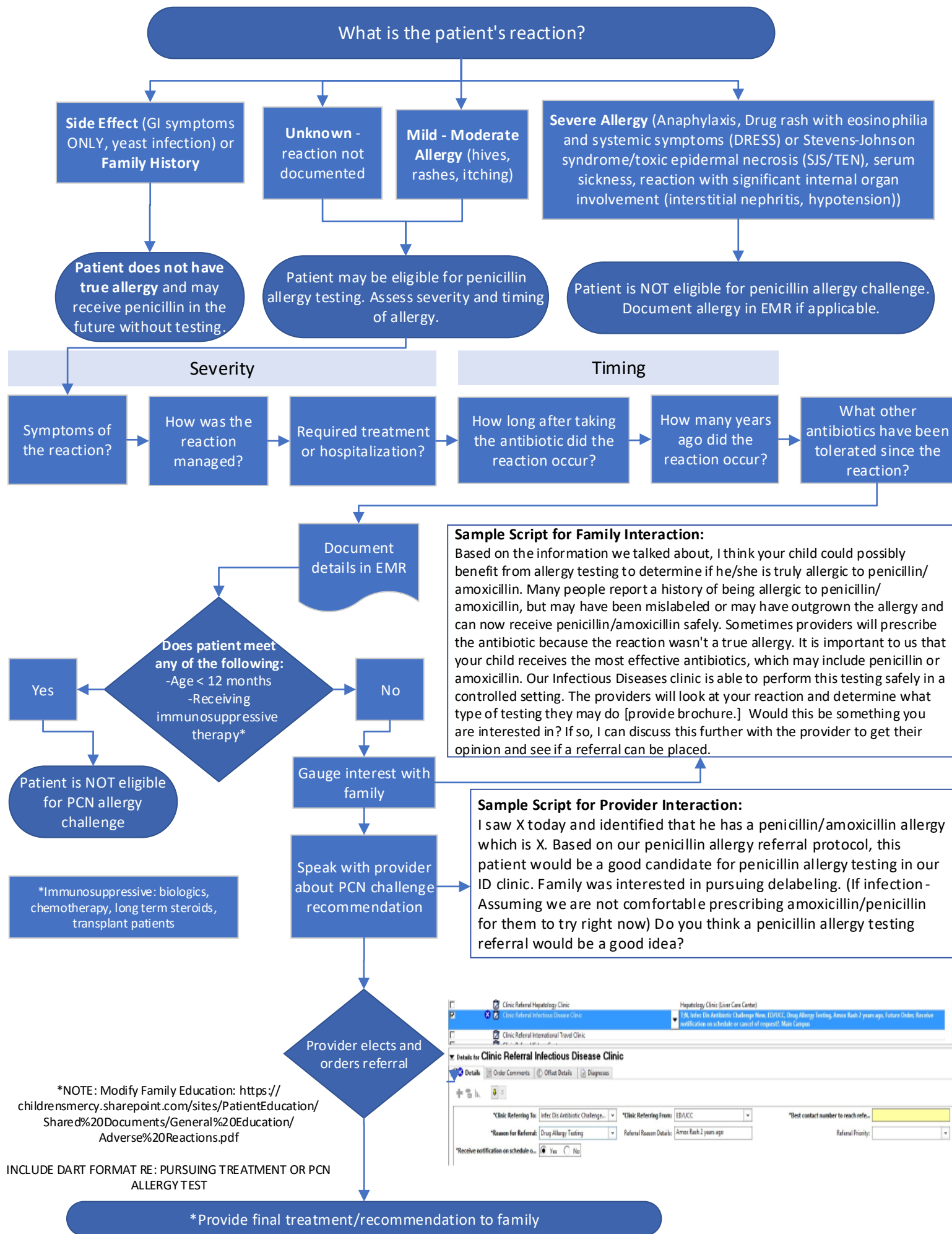

Supplement: Supplementary file 1 [file children-12-01087-s001.zip › children-3799270-supplementary.pdf]
